# Identification of Metabolomic Biomarkers of Long-Term Stress Using NMR Spectroscopy in a Diving Duck

**DOI:** 10.3390/metabo12040353

**Published:** 2022-04-15

**Authors:** Asha Perera, Catherine Soos, Karen Machin

**Affiliations:** 1 Department of Veterinary Pathology, Western College of Veterinary Medicine, University of Saskatchewan, 52 Campus Drive, Saskatoon, SK S7N 5B4, Canada; asha.perera@usask.ca; 2 Ecotoxicology & Wildlife Health Division, Science & Technology Branch, Environment and Climate Change Canada, 115 Perimeter Road, Saskatoon, SK S7N 0X4, Canada; catherine.soos@ec.gc.ca; 3 Department of Veterinary Biomedical Sciences, Western College of Veterinary Medicine, University of Saskatchewan, 52 Campus Drive, Saskatoon, SK S7N 5B4, Canada

**Keywords:** metabolomics, NMR spectroscopy, lesser scaup, stress physiology, corticosterone, hormone implants, energy metabolism

## Abstract

Human-induced environmental changes that act as long-term stressors pose significant impacts on wildlife health. Energy required for maintenance or other functions may be re-routed towards coping with stressors, ultimately resulting in fluctuations in metabolite levels associated with energy metabolism. While metabolomics approaches are used increasingly to study environmental stressors, its use in studying stress in birds is in its infancy. We implanted captive lesser scaup (*Aythya affinis*) with either a biodegradable corticosterone (CORT) pellet to mimic the effects of a prolonged stressor or a placebo pellet. 1D ^1^H nuclear magnetic resonance (NMR) spectroscopy was performed on serum samples collected over 20 days after implant surgery. We hypothesized that CORT pellet-induced physiological stress would alter energy metabolism and result in distinct metabolite profiles in ducks compared with placebo (control). Quantitative targeted metabolite analysis revealed that metabolites related to energy metabolism: glucose, formate, lactate, glutamine, 3-hydroxybutyrate, ethanolamine, indole-3- acetate, and threonine differentiated ducks with higher circulatory CORT from controls on day 2. These metabolites function as substrates or intermediates in metabolic pathways related to energy production affected by elevated serum CORT. The use of metabolomics shows promise as a novel tool to identify and characterize physiological responses to stressors in wild birds.

## 1. Introduction

The natural environment continues to change rapidly at unprecedented rates due to human activities globally. Habitat loss, pollution, climate change, and exposure to invasive exotic species remain as the main contributors for rapid environmental change [1]. Such long-term environmental changes could act as long-term stressors on free-ranging wildlife that rely on the environment for survival and reproduction. When exposed to a stressor, the hypothalamic–pituitary–adrenal axis redirects energy available for normal body maintenance functions to respond to and cope with the stressor [2]. While short-term stress responses promote immediate survival, long-term or chronic stress responses can be energetically expensive and have subsequent impacts on health, reproduction, and survival [2,3]. For example, long-term elevations in glucocorticoid (GC) stress hormones reduced growth and fledging success in tree swallow nestlings [4], reduced parental investment in penguins [5], increased self-maintenance behavior at the expense of reproductive behavior in barn owls [6], and compromised cognitive abilities during growth in kittiwakes [7]. Therefore, timely identification and quantification of the effects of long-term stressors are crucial to further understand population-level impacts on wildlife.

Gauging the magnitude of stress response of free-ranging animals is challenging [8]. An individual could face multiple stressors simultaneously in their natural environment, in comparison to a laboratory where environmental conditions can be controlled. In addition to the type, duration, and intensity of stressors, variability of the stress response depends on other critical factors such as prior experience, perception of a particular stress, genetic predisposition, condition, and age [9,10,11]. Therefore, an individual’s stress response is a cumulative result of genetic, metabolic, and environmental factors. Standard methods that are used to quantify a stress response, such as immunological and hormonal evaluations, typically measure a single compound and fail to connect the downstream biological effects to underlying physiological mechanisms [12]. Metabolite profiling techniques can provide a molecular-level understanding of the physiological mechanisms that link environmental stressors to stress responses and observed downstream changes in survival and reproduction.

Metabolomics is the study of small molecules within organs or organisms [13,14]. Nuclear magnetic resonance (NMR) spectrometry is one of the two analytical techniques used to assess significant deviations in the metabolism by characterizing the global metabolite profile [15]. Environmental metabolomics involves the use of metabolomic techniques to evaluate interactions between organisms and their environment [13,16,17]. The goal is to evaluate the metabolomic profiles of individuals in a population currently affected by a specific perturbation in order to identify a key set of metabolite biomarkers that are affected by the stressor(s). Screening for such metabolic biomarkers could, in turn, be used for early identification of exposure to similar perturbations before considerable disturbance occurs to populations or ecosystems, and ultimately to inform management decisions to mitigate, prevent, or minimize adverse effects, particularly to vulnerable populations [16,18]. 

The use of NMR-based metabolomics techniques to study stress in wild bird species has yet to be explored. Most avian metabolomics studies to date have focused on domestic birds in the food industry. A recent study on the avian metabolome in multiple tissues and biofluids of domestic chickens (*Gallus gallus domesticus*) provided a critically needed baseline database for researchers interested in the avian metabolome [19]. Other studies investigated the metabolome of domestic chickens in relation to feeding efficiency [20,21], meat flavour [22], and antibiotic growth promoters [23]. Recent studies have also examined the role of stress on metabolic alterations in multiple tissues and serum of chickens [24,25,26]. For example, chronic heat stress in broilers altered multiple pathways related to glucose, amino acid, and lipid metabolism in the liver [24]. In another study, the serum metabolome of heat-stressed broilers was used to understand mechanisms that caused poor growth performance [25]. Our goal was to evaluate the use of metabolomics approaches to identify key metabolomic biomarkers in response to chronic stress, using captive lesser scaup (*Aythya affinis*) as a model species in order to potentially inform studies on stress in wild birds.

The lesser scaup is a diving duck species that has experienced population declines in North America due to hunting, habitat loss, climate change, and contaminants [27]. Such prolonged, mostly anthropogenic stressors cause low survival and poor reproductive success [28] and are detrimental to individual and population health [29,30]. As the lesser scaup has experienced population declines due to multiple long-term stressors, it is an ideal model species in which to study stress responses. Understanding cellular level responses to stress via metabolomics will enhance the current understanding of the pathophysiology of stress and its consequences. To evaluate the use of metabolomics to study metabolite fluctuations in ducks undergoing stressful energy-demanding conditions, we used synthetic pellets containing corticosterone (CORT; the primary GC hormone associated with the stress response in birds) to simulate a stressful event (over several days) in captive lesser scaup. We performed NMR spectrometry on the serum to test the hypothesis that ducks experiencing chronic stress will have metabolite profiles consistent with increased energetic demands.

## 2. Results

### 2.1. Serum CORT

**Figure 1:**
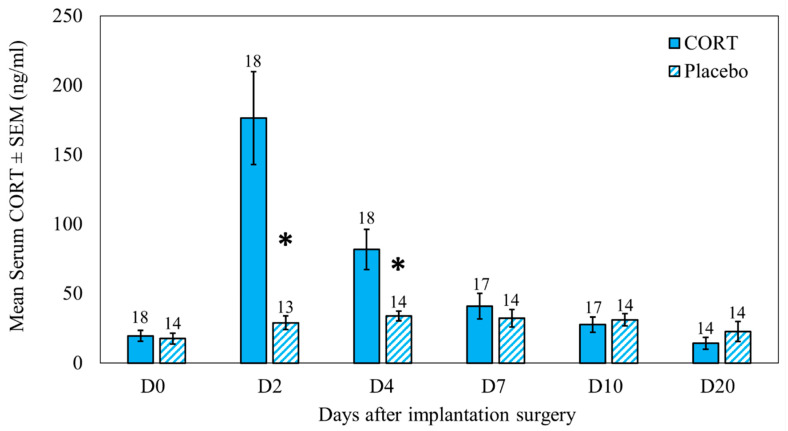
Serum CORT levels (mean ± SEM) before (D0), during (D2, D4), and after (D7, D10, D20) the active-pellet period from lesser scaup (*Aythya affinis*) that received either a 10-day slow-release cholesterol-based biodegradable CORT pellet (CORT) or placebo pellet that did not contain any hormone (control) on D0. Sample sizes are stated on each error bar. The asterisk (*) denotes statistical significance at *p* < 0.05.

Mean serum CORT and variability observed for each blood sampling day for treatment and control groups are shown in Figure 1. Fifteen CORT birds showed the same trend (highest CORT on D2 followed by a decrease on D4 and then a drop to preimplant (DO) levels towards D7). However, three implanted birds took up to 7 or 10 days for CORT levels to drop to D0 levels. 

In the general linear models (GLM) with repeated measures, we first explored CORT treatment and bleeding day as the main effects. We found CORT treatment (F_1,25_ = 10.42, *p* < 0.01) and bleeding day (F_5,125_ = 16.70, *p* < 0.01) were significant predictors of serum CORT. Therefore, we explored the interaction term in the model. The interaction between treatment and the bleeding day was significant (F_5,125_ = 20.58, *p* < 0.01). Thus, we analyzed the effect of treatment by bleeding day. There was no difference between groups immediately before implantation on D0 (*t* = −0.05, *p* = 0.96). Serum CORT results confirmed that synthetic CORT pellets actively released CORT into the circulation as treatment ducks had higher serum CORT values than controls on D2 (*t* = 3.90, *p* < 0.01) and D4 (*t* = 2.32, *p* = 0.03) (Figure 1). There were no differences between treatments at D7 (*t* = 0.96, *p* = 0.35), D10 (*t* = −1.39, *p* = 0.18), and D20 (*t* = −0.95, *p* = 0.35) (Figure 1), indicating that the CORT implant activity diminished to non-significant levels between D4 and D7, which was earlier than the manufacturer specified 10-day active period for CORT pellets. 

### 2.2. Serum Metabolites

Treatment and control groups consisted of 18 and 14 ducks, respectively. Metabolites were identified using the built-in metabolite identification system in Chenomx software and the human metabolome database (HMDB, https://hmdb.ca/ (accessed on 29 March 2019)) before the statistical analysis. We had 4, 5, 1, and 3 missing serum samples on D0, D2, D4, and D10, respectively, due to the inadequate serum volume that remained after performing radio-immunoassay for serum CORT.

**Figure 2:**
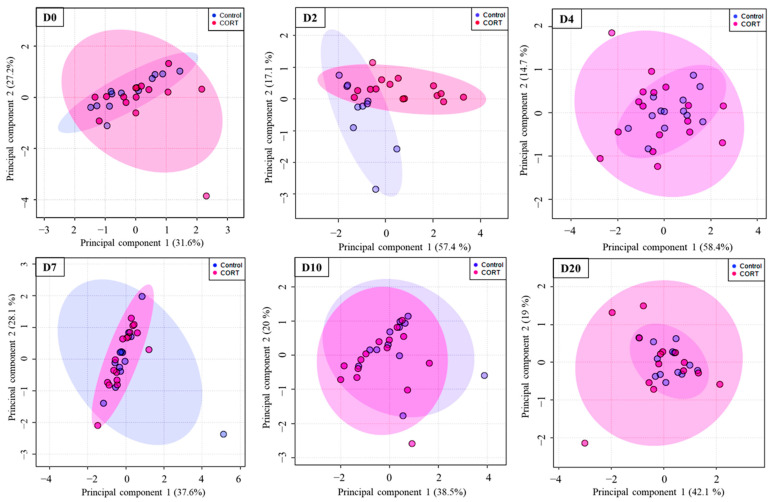
Principal component analysis (PCA) score plots of serum metabolites of lesser scaup (*Aythya affinis*) using data from before (D0), during (D2, D4), and after (D7, D10, D20) the active period of the implant. Pink and blue ellipses represent the 95% confidence region for CORT (treatment) and control, respectively. Overlapping 95% confidence regions indicate similarity between metabolite profiles in the two groups The separation of 95% confidence regions into clusters indicates a notable difference between metabolite profiles (D2). The percentage of variability explained under each principal component is shown within parenthesis on the x- and y-axes. Sample sizes on D0, D2, D4, D7, D10, and D20 were 28, 26, 31, 31, 28, and 28, respectively.

We profiled a total of 47 metabolites (Table 1) from 175 extracted serum samples of 32 lesser scaup from six sampling days. An unsupervised principal component analysis (PCA) showed that metabolite profiles overlapped between the treatment (CORT) and control (placebo) groups; thus, the two groups had similar metabolite profiles on all sampling days, except D2 (Figure 2). Metabolite profiles on D2 showed differentiation between the CORT and placebo groups on PCA (D2 on Figure 2). This robust clustering of metabolite profiles on D2 aligns well with the observed difference in serum CORT between the two groups on D2 (Figure 1). Metabolite profiles on D4 were not different between the two groups on PCA.

**Figure 3:**
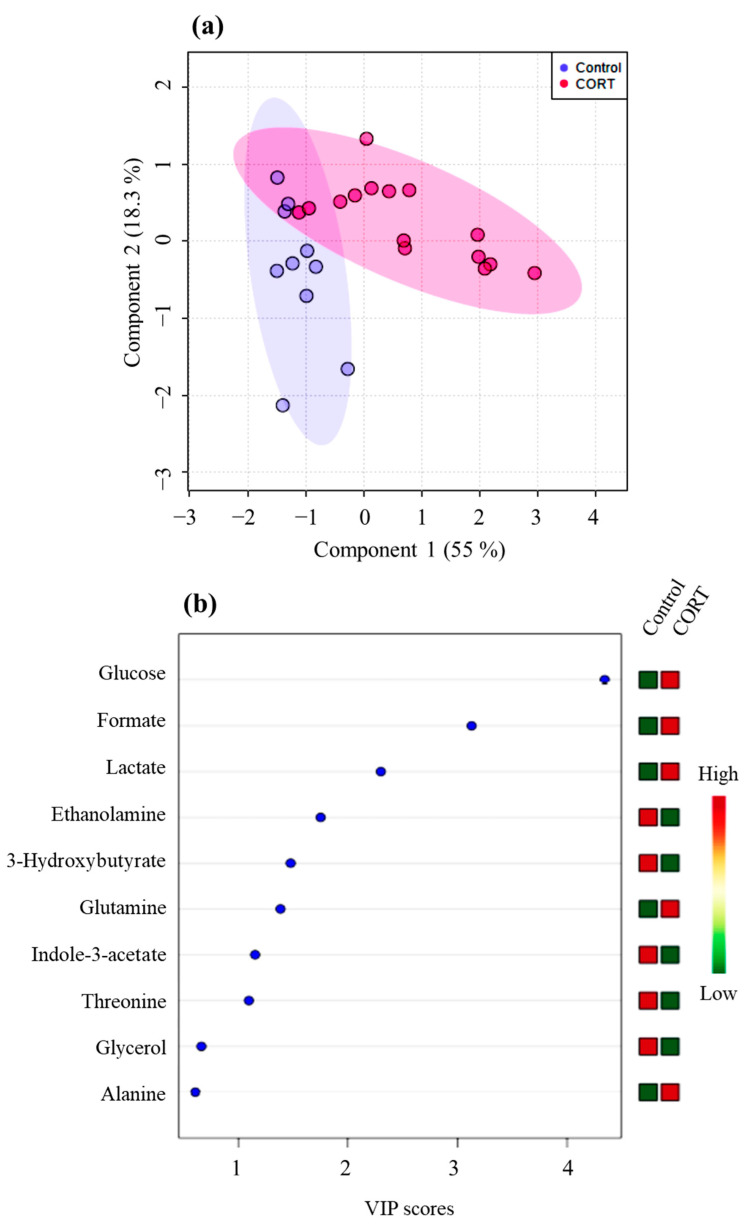
Partial least squares discriminant analysis (PLS-DA) scores plot (**a**) and corresponding variable importance in projection (VIP) score plot for D2 for component 1 (**b**) on D2 for serum of lesser scaup (*Aythya affinis*) that received either a CORT pellet (CORT) or a placebo (control). Numbers in parenthesis in axes of the PLS-DA score plot indicate the percentage of the variance explained by a corresponding component. In the VIP plot, metabolites with a VIP score > 1 are significant (i.e., VIP metabolites). The coloured boxes to the right of the VIP plot indicate which group had higher (red) vs. lower (green) levels of each metabolite compared to the other group.

**Figure 4:**
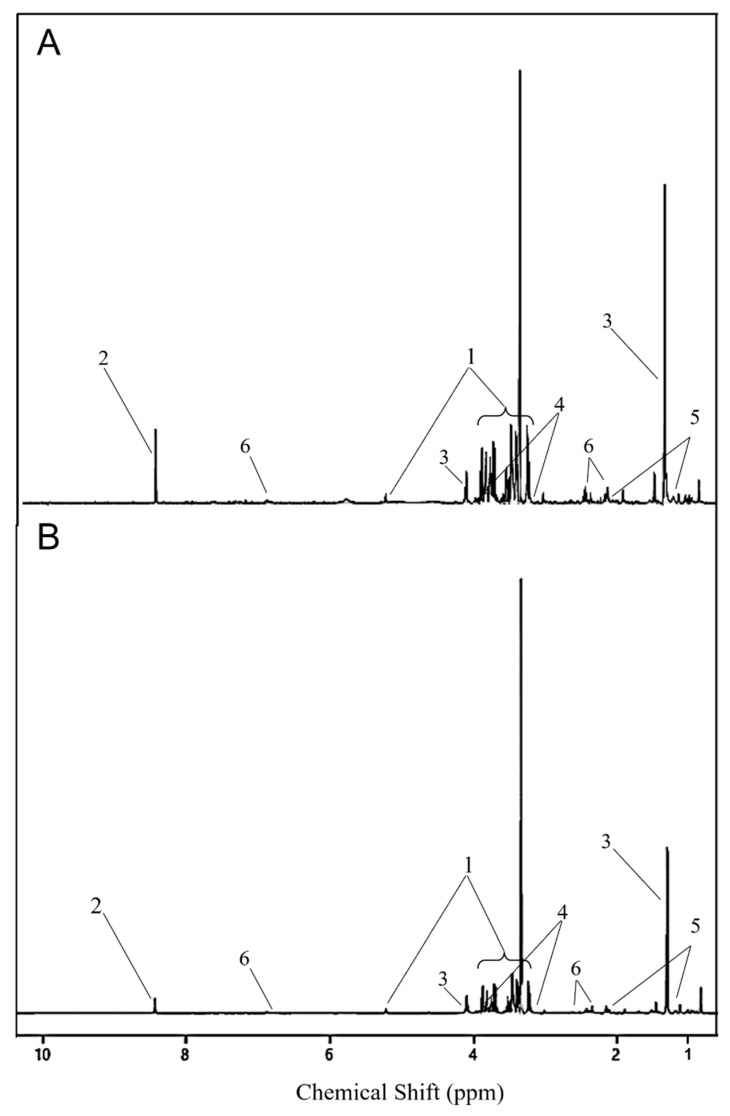
1D 600 MHz ^1^H NMR spectra from serum collected on D2 from lesser scaup (*Aythya affinis*) that received either a (**A**) 10-day slow-release cholesterol-based biodegradable CORT pellet (CORT) or a (**B**) placebo pellet. Same y-scale is used for both spectra. Metabolites with a significant corresponding variable importance in projection (VIP) score are shown: glucose (1), formate (2), lactate (3), 4-ethanolamine (4), 3-hydroxybutyrate (5), glutamine (6). Peaks of indole-3-acetate and threonine are not visible with this resolution of the spectra.

The partial least squares discriminant analysis (PLS-DA) model on metabolite data on D2 showed statistical significance with a 10-fold cross-validation (CV) value of 0.88 (accuracy), 0.67 (R2), and 0.52 (Q2), which validated the clustering seen on PCA (Figure 3a). This model also showed acceptable prediction accuracy using permutation tests (*p* = 0.01), indicating a significant difference between the metabolite profiles between CORT and control ducks on D2. The Variable Importance in Projection (VIP) metabolites that contributed to the significant difference in metabolite profiles on D2 were glucose, formate, lactate, ethanolamine, 3-hydroxybutyrate, glutamine, indole-3-acetate, and threonine (Figure 3b, Table 2). Glycerol and alanine had VIP scores <1, indicating that these two metabolites did not contribute to metabolite profile differences between the two groups. Glucose, formate, lactate, and glutamine were higher in CORT-implanted birds compared to control ducks, while ethanolamine, 3-hydroxybutyrate, indole-3-acetate, and threonine were lower in the CORT-implanted ducks compared to the control ducks. A schematic comparison of NMR spectra from a CORT-implanted duck and a control duck is shown in Figure 4. For D4, D7, D10, and D20, PLS-DA models were not significant (10-fold CV values < 0.6 and permutation test, *p* > 0.05; Figure S1). 

**Figure 5:**
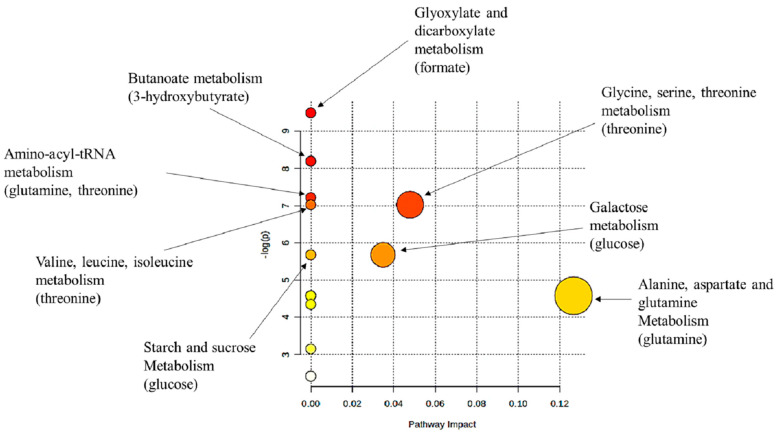
Pathway analysis plot based on serum metabolite profiles of analyses of lesser scaup (*Aythya affinis*), using the chicken (*Gallus gallus*) pathway library, showing significantly impacted pathways based on significant VIP metabolites that compare treatment (CORT-implanted) vs. control (placebo-implanted) groups. Only pathways that have impact scores > 0 from pathway topology analysis or significant Holm–Bonferroni adjusted *p*-values from pathway enrichment analysis are labelled. The diameter of each node corresponds to the impact score, and their colour gradient is related to the *p*-values (red = highly significant, white = non-significant).

The pathway analysis that was performed using the chicken (*Gallus gallus*) pathway library for all VIP metabolites revealed several key pathways of intracellular metabolism that were affected by elevated serum CORT (Figure 5). It is important to note that three of the eight VIP metabolites, namely, lactate, ethanolamine, and indole-3-acetate, were not accounted for in this analysis, likely due to the genetic, metabolic, and dietary differences in lesser scaup compared to chicken.

### 2.3. Body Weight

We weighed ducks on D0 before implant surgery, D10, and D20. The mean (SE) of weights for CORT ducks on D0, D10, and D20 were 607.78 (10.37) g, 563.44 (17.23) g, and 595.71 (22.94) g, respectively. The mean and SE of weights of control ducks on D0, D10, and D20 were 620.71 (13.11) g, 593.21 (8.15) g, and 604.64 (11.25) g, respectively. CORT and control ducks had similar body weights on all three sampling days: D0 (*t* = −0.78, *p* = 0.219), D10 (*t* = −1.49, *p* = 0.073), and D20 (*t* = −0.35, *p* = 0.365), where the negative weight trend in CORT ducks were notable on D10. 

## 3. Discussion

This experimental study demonstrates that ^1^H-NMR could capture the stress-induced metabolomic variation in ducks. We found that the metabolite profiles could differentiate treatment ducks from control when the serum CORT showed the highest difference between the two groups, on D2 post-surgery. Even though serum CORT levels suggested that CORT pellets were active for at least four and less than seven days post-implantation, metabolite profiles of CORT-implanted ducks were only different from controls on D2, when the serum CORT difference between the two groups was most pronounced, and CORT levels in the CORT-implanted ducks were highest. We identified a panel of key metabolites that differentiated the CORT-implanted ducks from the controls. High circulatory CORT was associated with elevated serum glucose, formate, lactate, and glutamine, and decreased serum ethanolamine, 3-hydroxybutyrate, indole-3-acetate, and threonine. To our knowledge, this is the first study to use NMR-based metabolomics to study stress responses in a wild duck species. 

We believe that the stress induced by the implantation procedure of the pellet had a negligible impact on the metabolic profile. The implantation procedure and duration of handling were similar for both treatment and control ducks. Thus, we expect both groups to experience similar stress related to handling and similar metabolite level effects for both groups, which will not affect the overall metabolite differences between the two groups. Furthermore, serum CORT in the placebo group remained the same as pre-implantation levels throughout the study, which indicates the effect was pellet implantation had a trivial effect on serum CORT and ultimately on metabolite profiles.

Short-term stressors (e.g., predation attempt, sudden severe storm, capture and handling) last for the time duration of minutes, whereas long-term stressors last within the time frame of days to months or longer (e.g., prolonged severe weather, human disturbance, change or loss in social status, habitat loss, chronic diseases) [31]. During short-term stressors, energy demands within the individual are not changed substantially because of the short duration of the stressor. Under the effects of the sympathetic nervous system and a brief increase of glucocorticoid hormones, energy mobilization is activated for a short duration that returns to pre-perturbation levels quickly. During short-term stress, GCs act to stimulate gluconeogenesis, glycogenolysis, and lipolysis [2,32]. In contrast, when faced with a long-term stressor, elevated energy demands are met at the expense of normal life functions to cope with the ongoing stressor, and an individual animal may end up in a negative energy balance that could be detrimental to their health and survival [31].

We found that glucose was the main metabolite contributing to the metabolite profile variation on D2. Given that glucose is the primary substrate used for cellular energy production, increases in glucose in response to prolonged elevations in circulatory CORT were not unexpected. While epinephrine and glucagon contribute to the initial increase in circulatory glucose during a stress response, GCs elicit a delayed secondary response to maintain elevated circulatory glucose levels [2]. Prolonged elevation of GCs stimulates gluconeogenesis via the catabolism of stored glycogen and synthesis of glucose from non-carbohydrate sources such as amino acids and glycerol [33]. In birds, which have higher plasma glucose levels compared to mammals of similar size, the shift to gluconeogenesis occurs more promptly than in mammals, due to the higher metabolism and low hepatic glycogen stores needed to maintain higher plasma glucose concentrations. In line with our findings, Jastrebski and colleagues subjected broilers to chronic heat stress for one week and found elevated liver glucose in stressed birds [24]. In contrast, Lu et al. [25] reported lower serum glucose concentration in broilers that experienced chronic heat stress for 14 days. The reduced feed intake was simultaneous with the heat stress for 14 days, likely resulting in a negative energy balance. 

Formate is produced intracellularly by oxidation of the non-essential amino acid serine within the cytoplasm or mitochondria [34]. The principal function of formate in cellular metabolism is to act as a carbon donor for purine nucleotide synthesis [35]. In energy metabolism, the role of formate is to produce adenosine nucleotides, adenosine monophosphate (AMP), or adenosine diphosphate (ADP) [36]. Under the influence of CORT, circulatory glucose is elevated. Then, glucose undergoes phosphorylation and glycolysis to produce adenosine triphosphate (ATP) to meet the energy demands. The conversion of ADP or AMP into ATP releases usable energy. Thus, a continued intracellular synthesis of formate as a carbon donor becomes crucial to meet the continued energy demands under the prolonged elevation of CORT, which explains why CORT-implanted ducks showed a marked rise in serum formate when serum CORT was the highest on D2. Concurrent with our findings, Zhu et al. [37] reported elevated urinary formate after long-term exposure to selenium in humans. In contrast to our findings, rats given prednisolone (a synthetic GC) injections for 12 weeks to induce GC-induced osteoporosis showed lower formate concentration than controls [38]. The reduction in formate concentration most probably resulted from abnormalities in the intestinal bacterial flora metabolism under the effect of long-term GC injections. In contrast, the relatively short duration of activity of our CORT pellets (<4 days) likely caused a comparatively smaller change in gut microbiome, and thus elevated formate synthesis aided in nucleotide and energy production. Gut microbiome investigation was out of the scope of this study.

Lactate was elevated in the CORT-treated ducks. Beers et al. [39] also reported elevated circulatory lactate in heat-stressed broilers. However, Jastrebski and colleagues [24] who used liver samples from 28 days post-hatch chicken did not report lactate as a contributor to metabolite variation between heat-stressed birds and controls. The activation of different metabolic pathways in a growing bird (compared to adult ducks used in our study), together with the complex metabolic pathway interactions in liver samples (in contrast with serum from our study) could have contributed to lactate being an insignificant metabolite in that study. Lactate was previously considered as the end-product of anaerobic glycolysis. However, more recently, the role of lactate was described as a crucial intermediate of aerobic metabolism [40]. Being a central carbon donor for the tricarboxylic acid (TCA) cycle via pyruvate production, lactate contributes to aerobic energy metabolism [41]. Furthermore, lactate functions as the most utilized gluconeogenic substrate in the avian liver [42]. Under the influence of prolonged elevations of circulatory CORT, lactate likely played a role as a gluconeogenic substrate. 

We saw an increase in glutamine but a decrease in threonine in response to prolonged increases in serum CORT. Glutamine is a non-essential amino acid that acts as a glucogenic precursor for the TCA cycle. The essential amino acid threonine contributes as a glucogenic and ketogenic contributor to the TCA cycle [43]. Furthermore, threonine contributes to the formate cycle for serine production [42]. Similar to our findings, Wang et al. [44] reported a reduction in threonine in rats that underwent mild, unpredictable, chronic stress. In contrast to our findings, the same study found reduced glutamine in chronically stressed rats. However, Lu et al. [25] reported increases in several amino acids (not glutamine) and the loss of breast muscle mass in chronically heat-stressed poultry, which indicated energy mobilization via protein degradation. Elevated GCs such as CORT reduce protein synthesis [45] and induce protein degradation in muscle [46] to supply gluconeogenic amino acids. While the reason behind simultaneous elevation in glutamine and reduction of threonine is unclear, we can deduce that elevated CORT had a profound impact on the amino acid metabolism in CORT-implanted ducks. The complex interplay of amino acids as precursors and intermediates in multiple pathways within the energy metabolism could have played a role in the observed increases or decreases of specific amino acids. 

Ketone bodies serve as alternative carbon sources of energy to help maintain ATP synthesis. Furthermore, ketone bodies act as functional molecules that reduce intracellular imbalances between oxidants and antioxidants by mediating gene transcription processes related to an oxidative damage response [47]. 3-Hydroxybutyrate (3-OHB) is a ketone body that is produced during periods of low circulatory glucose [46]. Ducks with CORT implants had lower levels of serum 3-OHB in the presence of elevated glucose, implying that 3-OHB was not acting as an alternative carbon source of energy during the active period of the implant. Multiple studies have reported noticeable rises in plasma 3-OHB in long-distance migrant birds [48,49,50]. 

Ethanolamine is a product of the hydrolysis of phospholipids [51]. It occurs as phosphatidyl-ethanolamine within cellular membranes, and as free ethanolamine in biofluids [51]. One of the main biologically essential functions of ethanolamine is the inhibition of mitochondrial respiration and reduction of aerobic respiration via the TCA cycle [52,53]. Under the influence of CORT, which promotes cellular aerobic respiration to enhance energy production, the ducks in this study experienced a reduction in ethanolamine concentration.

Indole-3-acetate is an end-product of microbial degradation of tryptophan. In line with our findings, Zheng et al. [54] reported lower indole-3-acetate in rats that experienced multiple mild unpredictable stressors for a prolonged time. They related the lower indole-3-acetate levels to reduced food consumption and therefore reduced intake of the essential amino acid tryptophan. Reduced feed intake is a well-known behavioural effect of chronic CORT administration in poultry [55,56,57]. In this study, CORT pellets were active in serum CORT for up to seven days. However, metabolite profiles were different between the treatment and control groups only on D2. Therefore, even though we saw an apparent reduction in body weight in CORT ducks on D10, it was not a lasting effect, possibly due to the comparatively shorter duration of the CORT activity observed in this study, after which birds were able to compensate.

We found that metabolite concentrations at D4 were similar between the two groups, even though the serum CORT was still higher in treatment ducks. Activation of the negative feedback mechanism after high serum CORT on D2 could have contributed to lower CORT on D4 [11]. This result further suggests that the serum CORT difference between groups should be considerably higher to observe differences in overall metabolite profiles related to energy metabolism (similar to D2). A stress response is a complex biochemical process that involves multiple molecular and organismal elements [58]. Elevated GCs are a major component of a stress response that primarily facilitates a shift in energy balance to deal with the stressor [58]. Metabolites are downstream functional components of a stress response. The fact that the metabolite profiles related to energy metabolism are similar on D4 implies the potential effect of biochemical processes other than energy metabolism on the shared metabolites that contribute to biochemical processes other than energy metabolism at lower elevations of GC concentrations. Our findings warrant the need for future studies to further investigate quantitative correlations between serum CORT and energy-related metabolite concentrations and to explore other metabolic pathways involved in a stress response. 

We demonstrated that NMR-based metabolomics could potentially play a role in studying the stress response in wild avian species. We report relevant patterns in several key serum metabolites related to energy metabolism, primarily caused by elevated circulatory CORT, under captive conditions in a wild diving duck species. Going forward, metabolomics techniques can be utilized to understand ecologically relevant causes and consequences of prolonged stress responses. We encourage future studies using this technique to explore potential sources of metabolite variations such as condition, food availability, climatic factors (e.g., temperature), and habitat quality, especially during energetically demanding phases of the annual life cycle of wild waterfowl (e.g., breeding and migration). Furthermore, metabolomics techniques could be used to investigate the downstream or carry-over effects on future condition, arrival time, reproductive success, or survival. The study of carry-over effects is ecologically important to further understand fitness consequences at the individual level or impacts on the population level. Metabolomics techniques have the potential to be used as an early screening tool to detect responses to environmental perturbations that may assist in identifying vulnerable populations in advance of harmful effects with conservation impacts. 

In summary, we report the potential of metabolomics as a novel tool to study the impacts of prolonged stress responses in a wild duck species. Using biodegradable CORT pellets in captive lesser scaup ducks, we demonstrated clear changes in metabolite profiles in response to prolonged elevations of CORT. Furthermore, we report the critical metabolites associated with energy metabolism that contributed to the observed differences in metabolite profiles between birds with high serum CORT and controls. Similarly, serum metabolomics can be used to identify wild bird populations that have been exposed to prolonged stressors. Future studies are required to link causes of stress to metabolite profiles and then to evaluate the individual and population-level impacts of stress. Metabolomics will continue to further enhance the current understanding of stress-related physiology and energy metabolism at a molecular level in wild birds. 

## 4. Materials and Methods

### 4.1. Study Area and Fieldwork

We used 32 adult captive lesser scaup raised from eggs collected from the wild. We housed all ducks in two fenced pens (8.5 m × 3.7 m each) constructed on the bank of a natural pond, approximately 10 km south of St. Denis, SK (52°06′ N, 106°04′ W, average elevation 561 m). Approximately half of each pen was water (height of water depended on pond water level, and the remainder allowed access to natural grass and shrubs on the shore of the pond). Pond water facilitated natural diving behaviour and provided access to natural aquatic invertebrate food sources. Ducks were supplied with a commercial poultry diet ad libitum. We followed the guidelines of the Canadian Council on Animal Care as defined by the Guide to the Care and Use of Experimental Animals. This project was approved by the University of Saskatchewan Animal Research Ethics Board—protocol no. 20030021.

### 4.2. Experimental Design

We conducted this experiment in parallel with another study that explored the relationship between serum CORT and feather CORT in lesser scaup during the natural moulting period (July–August) of lesser scaup. To simulate a chronic stress response and increase blood CORT, we utilized 10-day slow-release cholesterol-based biodegradable pellets (Innovative Research of America, Sarasota, FL, USA). The biodegradable CORT pellets generate a sustained circulatory release of CORT that can mimic a prolonged stress response [4,59]. In addition, these implants have been previously used successfully to elevate blood CORT in other avian species [4,60].

### 4.3. Surgical Procedure for Corticosterone Pellet Implantation

Each of the 32 ducks was randomly assigned to either the treatment group (n = 18; 11 females, 7 males) or the control group (n = 14; 9 females, 5 males). On day 0 (D0), ducks were subcutaneously implanted with either a pellet containing 42.5 mg CORT (treatment group) or a placebo pellet (control group), between shoulder blades at the base of the neck. We provided analgesia with a 2% lidocaine solution (2 mg/kg dose) injected subcutaneously at the selected surgical site, approximately 10 min before the skin incision. The mean mass (±SD) of ducks was 608 (±44) g and 621 (±41) g for CORT and control groups, respectively.

Briefly, we removed a few feathers from the surgical site and cleaned it using povidone-iodine solution (Betadine; Purdue Pharma, ON, Canada). A skin incision of ≈0.5 cm was made, followed by blunt dissection under the skin to make a small subcutaneous pocket. Sterile forceps were used to place a single pellet subcutaneously, and the skin incision was closed using tissue adhesive (Vet Bond; St. Paul, MN, USA). The surgical incision healed in all ducks within 2–3 days. 

### 4.4. Sample Collection and Storage

We collected blood samples (1–1.5 mL) on D0 (before CORT pellet implantation), D2, D4, D7, D10, and D20, from the right jugular vein within three minutes of entering the pen to minimize elevations in circulatory CORT associated with capture. Blood was transferred into serum vacutainers and placed in ice until processed. Samples were centrifuged within 2–3 h of collection, and serum was stored at −80 °C until laboratory analyses. Serum was analyzed for CORT and small metabolites related to energy metabolism using a radioimmunoassay and 1D ^1^H NMR spectrometry, respectively. We weighed all ducks on D0, before pellet surgery; D10; and D20. 

### 4.5. Radioimmunoassay for Serum Corticosterone

We analyzed serum samples for CORT using a commercially available radioimmunoassay (RIA) kit (ImmuChem double-antibody corticosterone ^125^I RIA kit, MP Biomedicals, NY, USA) at the Endocrinology lab, Western College of Veterinary Medicine, University of Saskatchewan, Canada. This kit has been previously used in multiple avian studies [60,61]. We followed the manufacturer’s protocol with modifications to assess serum CORT in ducks. Briefly, serum samples were diluted 1:20 with dilution solution (phosphosaline gelatin buffer, pH 7.0 ± 0.1) provided with the commercial kit. Aliquots of 100 µL of samples and standards were used in duplicate. First, we pipetted 0.18 mL of ^125^I- labelled CORT into all tubes. Then, 0.18 mL of antibody was added to all tubes except total count and non-specific binding tubes. After a 2 h room temperature incubation, we added 0.46 mL of precipitation solution to all tubes, except the total count tubes. All tubes were centrifuged at 3000 rpm for 40 min. Finally, we quantified the radioactivity of all tubes using a gamma counter. We performed RIA for duplicates of all serum samples in three RIA assays. As a measure of assay viability, the percent coefficient of variation was calculated using mean and standard deviation CORT measurement for the high and low standard CORT solutions provided in the kit. Intra-assay variation was 5.08 % (range 2.99–7.09), and inter-assay variation was 6.71%.

### 4.6. Sample Extraction and Preparation for NMR

We extracted the polar metabolites in serum using a methanol–chloroform protocol for extraction, modified from protocol 3.1.3 for tissue samples following Viant [62]. Briefly, serum samples that were stored frozen at −80 °C were thawed in a crushed ice bath. Then, 100 μL of serum from each sample was pipetted into a glass vial (height 45 mm × diameter 15 mm) in ice. After adding 400 μL ice-cold methanol and 60 μL ice-cold deionized water to the serum, each glass vial was vortexed for 10 s. We calculated the volume of deionized water volume for serum following Note 4: Monophasic solution proportions in Viant [62], assuming 100 μL of serum was equal to 100 μL of deionized water. After adding 200 μL of ice-cold chloroform for NMR, we vortexed each vial for 30 s. Vials were vortexed thoroughly for 10 min at 300 rpm to obtain a monophasic solution that contained methanol–chloroform–water at a 2:1:0.8 ratio. All vials were centrifuged at 1800× *g* for 5 min at 4 °C. We transferred the supernatant of each vial to a new glass vial (height 45 mm × diameter 15 mm) and added 200 μL of ice-cold chloroform for NMR and 200 μL of ice-cold deionized water. Each glass vial was vortexed for 30 s to prepare a biphasic solution with methanol–chloroform–water ratio of 2:2:1.8. Between each pipetting and vortexing step, we kept glass vials with serum and reagents in ice to minimize the error caused by metabolization of biological compounds in serum samples that could occur at room temperature. We centrifuged samples at 1800× *g* for 10 min at 4 °C and separated the solution into a methanol–water phase on the top and a chloroform phase at the bottom. The methanol–water phase contained polar metabolites, while the chloroform phase contained the non-polar and lipophilic compounds. We carefully pipetted the upper and lower layers of each sample into separate glass vials using glass Pasteur pipets. Finally, we freeze-dried all polar and non-polar extracts and stored them at −80 °C. For NMR, we used the methanol–water phase that contained polar metabolites. We re-constituted the dried polar fraction in 200 μL of 0.1M NMR buffer that was prepared following Section 3.1.4 in Viant [62]. NMR buffer contained a sodium phosphate buffer (pH 7.0, composed of NaH_2_PO_4_ and Na_2_HPO_4_ salts, Thermo Fisher Scientific, MA, USA) made up in D_2_O (99.9% purity; Goss Scientific Instruments, Great Baddon, UK), containing 0.5 mM sodium 3-trimethylsilyl propionate-2,2,3,3-d_4_ (TMSP) as the internal chemical standard. Then, samples were vortex-mixed for 10 s. We centrifuged samples at 12,000× *g* for 5 min at room temperature and transferred the supernatant to 3 mm OD stem NMR tubes (NE-H5/3-Br, New Era Enterprises, Inc., Vineland, NJ, USA). We performed NMR on the same day after transferring samples to stem NMR tubes.4.7. 1D ^1^H-Nuclear Magnetic Resonance.

NMR spectra were acquired for polar serum extracts on a Bruker Avance III HD spectrometer operating at 600.17 MHz, equipped with a 5.0 mm BBO probe (298 K). All ^1^H-NMR spectra were the average of 128 transients acquired with 32,768 points, an acquisition time of 1.7 s, and a repetition delay of 2 s between transients. The pulse sequence was zgesgp. The TMSP peak was used to reference the chemical shifts of polar extracts. Concentrations of metabolites in the polar fraction were measured relative to that of the TMSP peak in each NMR spectrum. Bruker Topspin 3.2 software (Bruker Corporation, Billerica, MA, USA) was used to manually baseline and phase-correct spectra by a single operator. The spectral width was 10 ppm.

### 4.7. Data Processing and Statistical Analysis

#### 4.7.1. Effect of CORT Treatment on Serum CORT

We performed GLM for repeated measures using SPSS software (Version 25, International Business Machines Corp., Armonk, NY, USA). First, we used pen, sex, and treatment (CORT or placebo) as predictor variables and day as the repeated measure in univariate models to determine any effects on serum CORT. Pen (F_1,25_ = 0.06, *p* = 0.80) and sex (F_1,25_ = 0.6, *p* = 0.45) had no effect on serum CORT and thus were not included in subsequent models. To assess the CORT treatment effect on serum CORT, we used treatment as the predictor variable and serum CORT on different days as the repeated measure. We excluded five ducks (four CORT and one control) from the repeated measures analysis because they had missing serum CORT values for one or more days. 

#### 4.7.2. Serum Metabolites

A list of metabolites related to stress responses and related energy metabolism was compiled using previously published literature. Chenomx NMR Suite 8.3 software (Chenomx Inc., Edmonton, AB, Canada) was used to process each spectrum (Chenomx Processor software) to identify each peak (Chenomx profiler software). We assigned spectral peaks to individual metabolites by comparing chemical shifts of 1D ^1^H-NMR spectra from samples to metabolites available at the Chenomx Compound Library. Metabolite profiling for all samples (n = 175) was performed by a single person (A.P.) using the metabolite fitting algorithm available within the Chenomx software. 

We exported the compound concentrations into separate Excel (Microsoft, WA, USA) datasheets for each sampling day. Then, we uploaded each metabolite concentration datasheet to MetaboAnalyst 4.0 software (www.metaboanalyst.ca (accessed on 13 February 2022)) [63]. We did not perform a time-series analysis because only 17 (eight CORT birds, nine control birds) out of 32 birds had metabolite data available for all six sampling days. We identified outliers as the samples that fell outside Hotelling’s *T^2^* ellipse and those were removed. The number of samples available for NMR analysis on each day differed due to failed bleeding attempts, death of four CORT ducks during the study period, and availability of inadequate blood volume after performing serum CORT RIA. We identified 58 metabolites in lesser scaup serum initially. However, after quantification, 11 metabolites had 57–92% missing values (percentage varied depending on the metabolite). Missing values could arise due to the absence of the corresponding spectral peak in a particular sample, the intensity of a peak being below the analytical precision [63]. Following the “80% rule” that recommends the exclusion of metabolites that contain missing values for >20% of samples [64,65], we excluded these 11 metabolites to obtain the final dataset that contained 47 metabolites. Spectra were row-wise normalized compared to the sample median and were Pareto-scaled. Pareto scaling was used because Pareto scaling provided the most normalized data closest to a bell curve.

We performed a PCA to investigate any difference in metabolite profiles between CORT and control groups. After clustering of the treatment groups was observed in PCA, we performed a PLS-DA to assess the significance of PCA clustering. PLS-DA models were validated using (i) 10-fold CV that used accuracy and R^2^ and Q^2^ values, and (ii) permutation tests that used prediction accuracy after 100 iterations. When 10-fold CV values > 0.6 and permutation test *p* < 0.05, PLS-DA models are considered accurate and predictive. Variable importance in projection (VIP) was used on the validated models to identify the metabolites that contribute significantly to discriminate the projected PLS-DA scores. The metabolites with VIP scores > 1 were considered significant. All statistical analyses were conducted in MetaboAnalyst 4.0. The significance level was set at *p* ≤ 0.05.

Pathway analysis was performed using the Pathway Analysis tool in MetaboAnlayst on significant VIP metabolites (hereafter, VIP metabolites) to identify the metabolic pathways that were differentially affected by CORT and control groups [66]. A concentration table that contained the VIP metabolite concentrations in columns and sample IDs in rows in Excel was used for pathway analysis. We used the chicken (*Gallus gallus*) pathway in MetaboAnalyst with a global test that used relative-betweenness centrality. We identified VIP metabolite concentrations using the corresponding human metabolome database (HMDB) identification numbers, and metabolite concentrations were normalized using Pareto scaling. In Pathway analysis, metabolite pathways that have impact scores > 0 and Holm–Bonferroni adjusted *p*-values ≤ 0.05 are considered significant pathways. 

#### 4.7.3. Body Weight

An independent samples *t*-test was used to compare means of body weights on D0, D10, and D20 between CORT and control groups.

## Figures and Tables

**Table 1:** Chemical shift and multiplicity of the metabolites (putatively annotated compounds using Chenomx NMR Suite 8.3 software) of 1D ^1^H NMR spectra of the polar extract of serum of lesser scaup (*Aythya affinis*) implanted with CORT or placebo pellets.

No.	Compound Name	^1^H NMR Chemical Shift (ppm) ^a^, Multiplicity ^b^
1	2-Hydroxybutyrate	0.88 t, 1.64 m, 1.73 m, 4.00 q
2	3-Hydroxybutyrate	1.19 d, 2.30 q, 2.40 q, 4.14 m
3	Acetate	1.91 s
4	Acetoacetate	2.27 s, 3.44 s
5	Alanine	1.47 d, 3.78 q
6	Anserine	2.65 m, 2.71 m, 3.05 q, 3.2 m, 3.78 s, 4.5q, 7.25 s, 7.92 s, 8.27 d
7	Arginine	1.64 m, 1.70 m, 1.88 m, 1.92 m, 3.24 t, 3.76 t
8	Asparagine	2.85 q, 2.94 q, 3.99 q, 6.91 s
9	Betaine	3.26 s, 3.89 s
10	Carnitine	2.41m, 3.21s, 3.42 m, 4.56 s
11	Choline	3.19 s, 3.51 t, 4.06 s
12	Citrate	2.53 d, 2.66 d
13	Creatine	3.02 s, 3.92 s
14	Creatinine	3.03 s, 4.05 s
15	Dimethylamine	2.72 s
16	Ethanolamine	3.13 t, 3.82 t
17	Formate	8.44 s
18	Fumarate	6.51 s
19	Glucose	3.24 t, 3.40 m, 3.51 m, 3.70 m, 3.82 m, 3.89 d, 4.64 d, 5.22 d
20	Glutamate	2.04 m, 2.12 m, 2.34 m, 3.75 m
21	Glutamine	2.13 m, 2.44 m, 3.77 t, 6.87 s
22	Glycerol	3.55 q, 3.65 q, 3.78 m
23	Glycine	3.55 s
24	Glycolate	3.93 s
25	Histamine	3.00 t, 3.29 t, 7.14 s, 7.89 s
26	Histidine	3.14 q, 3.24 q, 3.98 q, 7.10 s, 7.87 s
27	Imidazole	7.31 s, 8.28 s
28	Indole-3-acetate	3.64 s, 7.15 t, 7.24 t, 7.50 d, 7.62 d, 9.95 s
29	Isocitrate	2.50 q, 2.55 q, 2.98 m, 4.05 d
30	Isoleucine	0.93 t, 1.00 d, 1.25 m, 1.46 m, 1.97 m, 3.66 d
31	Lactate	1.32 d, 4.10 q
32	Leucine	0.95 t, 1.70 m, 3.73 q
33	Methionine	2.10 t, 2.13 s, 2.19 m, 2.63 t, 3.85 q
34	Phenylalanine	3.12 q, 3.28 q, 3.99 q, 7.32 d, 7.37 t, 7.42 t
35	Proline	1.96 m, 2.02 m, 2.06 m, 2.34 m, 3.32 m, 3.42 m, 4.12 q
36	Pyruvate	2.36 s
37	Sarcosine	2.7 s, 3.60 s
38	Serine	3.84 q, 3.94 q, 3.98 q
39	Succinate	2.39 s
40	Taurine	3.25 t, 3.42 t
41	Threonine	1.32 d, 3.58 d, 4.25 m
42	Trimethylamine N-oxide	3.25 s
43	Tryptophan	3.30q, 3.48 q, 4.05 q, 7.19 t, 7.27 t, 7.32 s, 7.53 d, 7.72 d
44	Tyrosine	3.04 q, 3.19 q, 3.93 q, 6.89 d, 7.18 d
45	Uracil	5.80 d, 7.53 d
46	Valine	0.98d, 1.03 d, 2.26 m, 3.60 d
47	Myo-inositol	3.27 t, 3.53 q, 3.62 t, 4.06 t

^a^ The NMR spectral position and number of chemical shifts of a compound determine the chemical structure. ^b^ Multiplicity is related to the number of hydrogens located in the neighbouring carbon in the chemical structure of a compound. s, singlet; d, doublet; t, triplet; q, quadruplet; m, multiplet.

**Table 2:** Mean and standard error mean (SE) for concentrations (mM) for serum polar metabolites of lesser scaup (*Aythya affinis*) that were identified to have variable importance in projection (VIP) score > 1, using PLS-DA modeling for D2.

VIP Metabolite (D2)	CORT		Control		*t* ^a^	*p* ^b^
	Mean (mM)	SE	Mean (mM)	SE		
3-Hydroxybutyrate	0.09	0.01	0.16	0.02	−4.07	<0.01
Ethanolamine	0.18	0.02	0.35	0.03	−2.14	0.04
Formate	0.48	0.08	0.16	0.21	4.81	<0.01
Glucose	3.29	0.29	2.53	0.19	3.26	<0.01
Glutamine	0.24	0.04	0.15	0.10	2.79	0.01
Indole-3-acetate	0.06	0.01	0.12	0.02	−2.69	0.01
Lactate	2.83	0.22	2.48	0.14	1.78	0.04
Threonine	0.14	0.01	0.18	0.02	−3.81	<0.01

^a,b^*t* and *p* correspond to the independent *t*-test results for mean comparison between the groups that received either a CORT pellet (CORT) or a placebo (Control), respectively. Statistical significance was set at *p* < 0.05.

## Data Availability

The data presented in this study are available upon request. Contact the corresponding author for details.

## Supplementary Materials

The following supporting information can be downloaded at https://www.mdpi.com/article/10.3390/metabo12040353/s1, Figure S1: Partial least squares discriminant analysis (PLS-DA) score plots for D4, D7, D10, and D20 for component 1 for serum of lesser scaup (*Aythya affinis*) that received either a CORT pellet (CORT) or a placebo (control).
