# Supplementary material for: Identification of Metabolomic Biomarkers of Long-Term Stress Using NMR Spectroscopy in a Diving Duck
Source: Metabolites. 2022 Apr 15;12(4):353. doi: 10.3390/metabo12040353 (PMC9030486; doi:10.3390/metabo12040353)
Supplement: Supplementary file 1 [file metabolites-12-00353-s001.zip › metabolites-1618322-supplementary.pdf]

Supplementary Materials

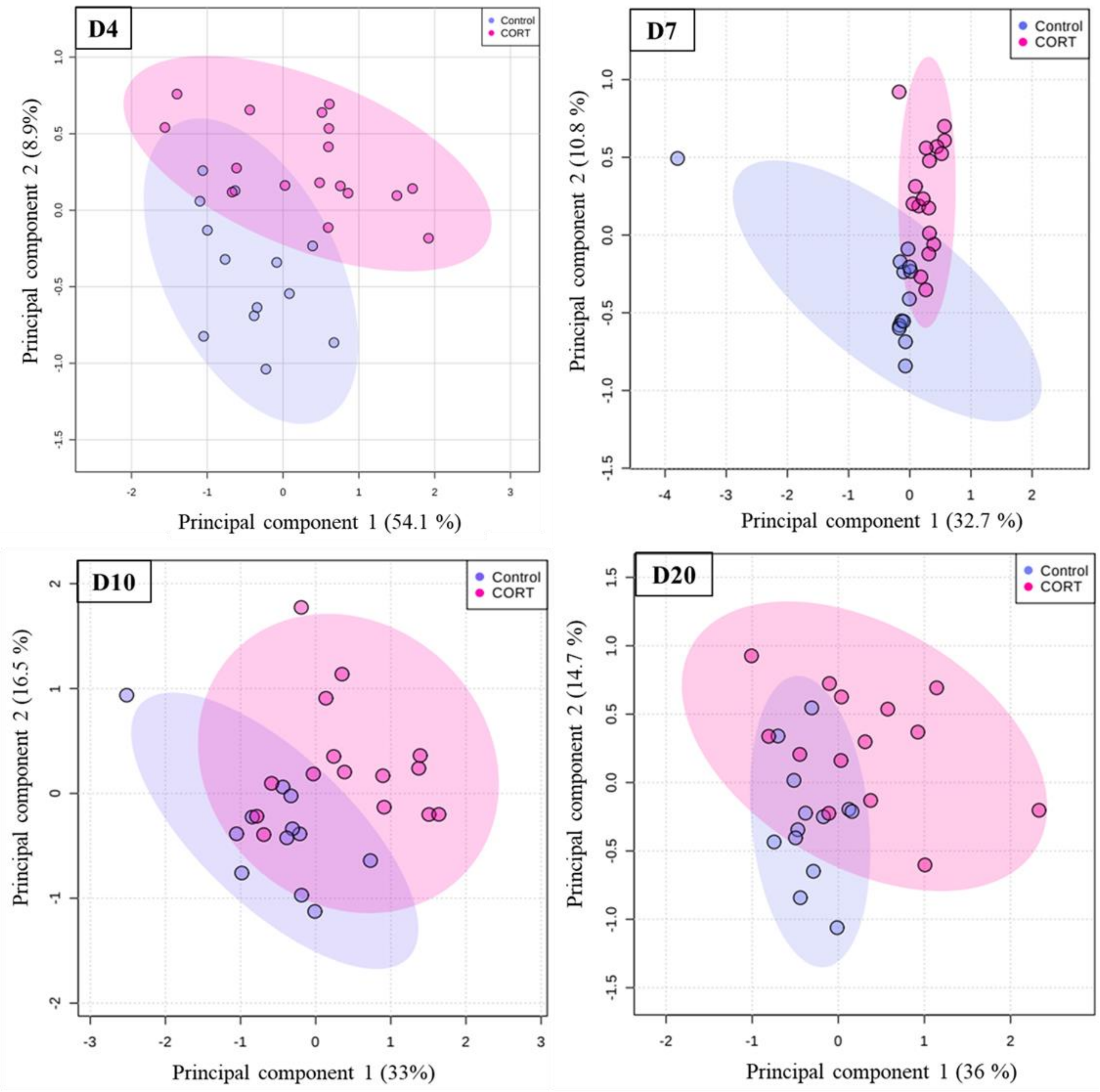

Figure S1. Partial least squares discriminant analysis (PLS-DA) scores plots for D4, D7, D10 and D20 for component 1 for serum of lesser scaup (*Aythya affinis*) that received either a CORT pellet (CORT) or a placebo (control). Numbers in parenthesis in axes of the PLS-DA score plot indicate the percentage of the variance explained by a corresponding component. For D4, D7, D10 and D20, PLS-DA models were not significant (10-fold CV values < 0.6 and permutation test $p > 0.05$).
